# Datasets for benchmarking antimicrobial resistance genes in bacterial metagenomic and whole genome sequencing

**DOI:** 10.1038/s41597-022-01463-7

**Published:** 2022-06-15

**Authors:** Amogelang R. Raphenya, James Robertson, Casper Jamin, Leonardo de Oliveira Martins, Finlay Maguire, Andrew G. McArthur, John P. Hays

**Affiliations:** 1 David Braley Centre for Antibiotic Discovery, McMaster University, Hamilton, Ontario L8S 4K1 Canada; 2 Michael G. DeGroote Institute for Infectious Disease Research, McMaster University, Hamilton, Ontario L8S 4K1 Canada; 3 Department of Biochemistry and Biomedical Sciences, McMaster University, Hamilton, Ontario L8S 4K1 Canada; 4 National Microbiology Laboratory, Public Health Agency of Canada, Guelph, Ontario N1G 3W4 Canada; 5 Department of Medical Microbiology, Care and Public Health Research Institute (CAPHRI), Maastricht University Medical Center, P. Debyelaan 25, 6229HX Maastricht, the Netherlands; 6 Quadram Institute Bioscience, Norwich Research Park, Norwich, NR4 7UQ UK; 7 Department of Community Health & Epidemiology, Dalhousie University, Halifax, Nova Scotia B3H 4R2 Canada; 8 Faculty of Computer Science, Dalhousie University, Halifax, Nova Scotia B3H 4R2 Canada; 9 Shared Hospital Laboratory, Sunnybrook Health Sciences Centre, Toronto, Ontario M4N 3M5 Canada; 10 Department of Medical Microbiology & Infectious Diseases, Erasmus University Medical Centre Rotterdam (Erasmus MC), Doctor Molewaterplein 40, 3015 GD Rotterdam, the Netherlands

**Keywords:** Bacterial infection, Infectious diseases

## Abstract

Whole genome sequencing (WGS) is a key tool in identifying and characterising disease-associated bacteria across clinical, agricultural, and environmental contexts. One increasingly common use of genomic and metagenomic sequencing is in identifying the type and range of antimicrobial resistance (AMR) genes present in bacterial isolates in order to make predictions regarding their AMR phenotype. However, there are a large number of alternative bioinformatics software and pipelines available, which can lead to dissimilar results. It is, therefore, vital that researchers carefully evaluate their genomic and metagenomic AMR analysis methods using a common dataset. To this end, as part of the Microbial Bioinformatics Hackathon and Workshop 2021, a ‘gold standard’ reference genomic and simulated metagenomic dataset was generated containing raw sequence reads mapped against their corresponding reference genome from a range of 174 potentially pathogenic bacteria. These datasets and their accompanying metadata are freely available for use in benchmarking studies of bacteria and their antimicrobial resistance genes and will help improve tool development for the identification of AMR genes in complex samples.

## Abstract (summary)

**Table:** 

Measurement(s)	bacterial genomes
Technology Type(s)	next generation DNA sequencing
Factor Type(s)	None
Sample Characteristic - Organism	Bacterium
Sample Characteristic - Environment	Varying
Sample Characteristic - Location	World

## Background & Summary

Whole genome sequencing (WGS) is a technique used to analyse the genomes of both prokaryotic and eukaryotic organisms. This includes a range of approaches including WGS of individual isolates (either via culture or single-cell methods) and the related simultaneous sequencing of all organisms present in a given sample (i.e., metagenomics)^1^. There are also a range of different sequencing technologies available such as technologies that generate ‘short-read’ or ‘long-read’ sequences^2^. Within the field of microbiology, sequencing is a valuable tool for mapping the epidemiology of bacterial isolates associated with clinical outbreaks of disease^3^, as well as for the identification of potentially pathogenic strains of bacteria that could be present in both food and environmental samples^4^. It is increasingly common to use sequencing to identify the type and range of antimicrobial resistance (AMR) genes present in bacterial isolates in order to make predictions regarding the actual bacterial phenotype of particular isolates^5,6^. These data have the potential to guide antibiotic treatment decisions and patient therapy in clinical cases of disease^7^. However, many different bioinformatic software and pipelines exist to predict AMR genes in genomic and metagenomic sequencing data. These include methods designed to directly analyse unassembled short and long-reads as well as those involving the assembly of these reads into contiguous bacterial chromosomes, partial chromosomes (contigs) and/or mobile genetic elements, such as plasmids^8–10^. The ability to systematically compare and benchmark the range of WGS algorithms and pipelines available on a common dataset would provide increased confidence in the validity of interpreting the results of WGS genotyping, AMR carriage, and the inferred bacterial AMR phenotype^11–13^. Such benchmarking activities would be promoted by the availability of common gold standard reference datasets containing raw sequencing reads, contigs, chromosomes, and plasmid data^14^ and including software associated with the assembly of both short and long-read sequence results^15^. Such a gold standard reference set of bacterial WGS data (focussing on short read sequence data and including simulated metagenomic data) was generated during the Microbial Bioinformatics Hackathon and Workshop 2021, which took place virtually between the 11^th^ and 13^th^ October, 2021. The event was jointly organized by the Public Health Alliance for Genomic Epidemiology (PHA4GE), the Joint Programming Initiative on Antimicrobial Resistance (JPIAMR), and the Cloud Infrastructure for Big Data Microbial Bioinformatics (CLIMB-BIG-DATA) initiative^16^.

## Methods

A selection of benchmarking genomes was made by prioritizing ESKAPE pathogens (i.e., *Enterococcus faecium*, *Staphylococcus aureus*, *Klebsiella pneumoniae*, *Acinetobacter baumannii*, *Pseudomonas aeruginosa*, and *Enterobacter* spp.) in addition to *Salmonella* spp. We selected only complete genomes from the NCBI Database Repository for Genome Access^17^, where the primary sequence data was available and the Illumina data deposited included >40X coverage and >100 bp sequence read length.

**Fig. 1:**
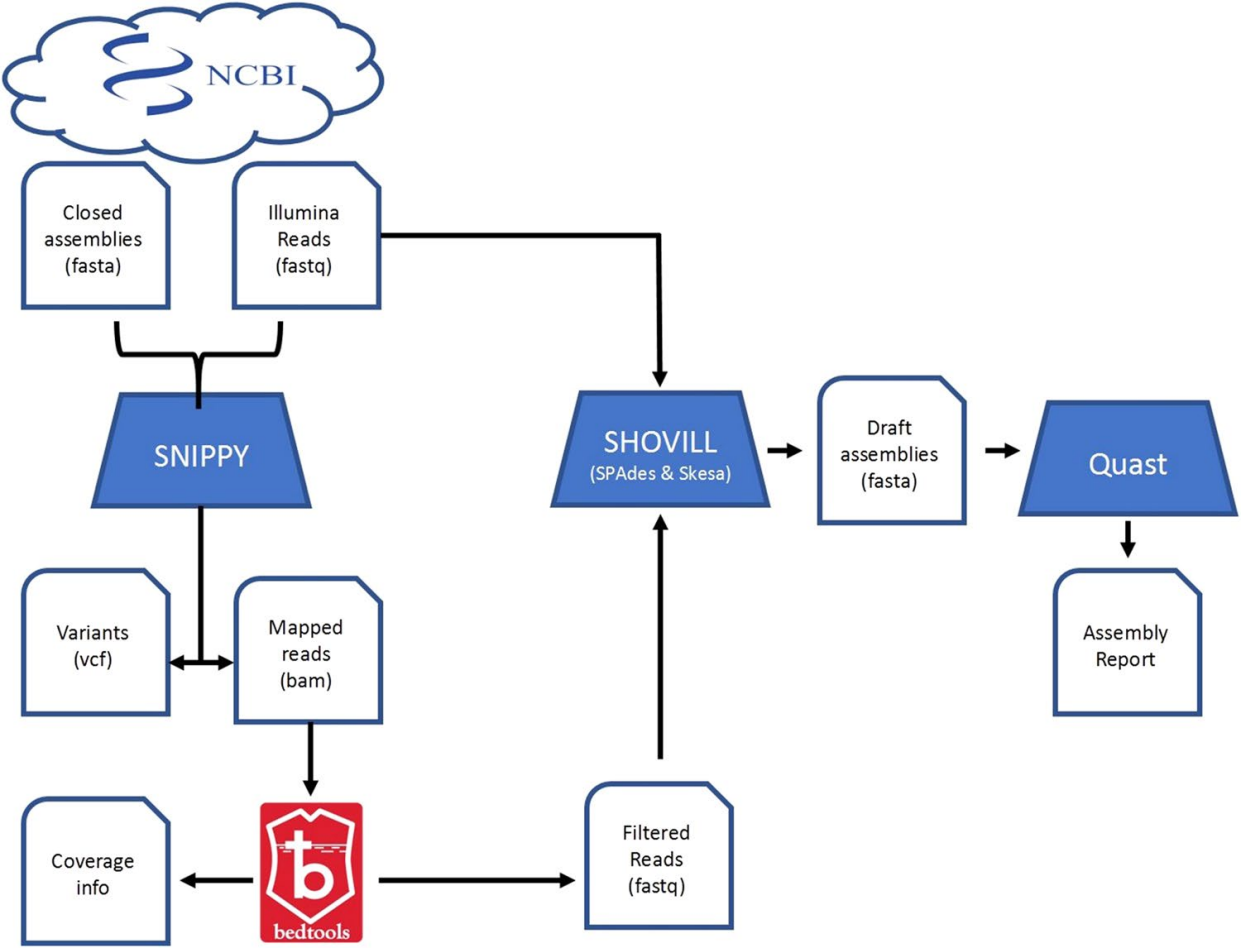
Diagram illustrating the sequence of steps and software involved in generating ‘gold standard’ bacterial whole genome sequence datasets for benchmarking bacterial assembly and prediction software.

Candidate genomes were processed using the workflow depicted in Fig. 1, with the genomes filtered according to the criteria described below. Initially, Illumina read sets were downloaded from NCBI and assembled using shovill v. 1.1.0^18^ using both SPades^19^ and Skesa^20^. Assembly metrics were determined using Quast v. 5.0.2^21^ and assemblies with N50 <50Kb and >100 contigs were excluded. Illumina reads were mapped against their corresponding NCBI genome using SNIPPY v. 4.3.6^22^ using the default parameters (minimum coverage depth = 10, minimum VCF quality = 100, minimum fraction = auto). Regions of 0 read coverage were identified using bedtools v. 2.29.2^23^ and genomes with >200Kb of no Illumina read coverage were excluded. Additionally, any samples where there were >10 SNPs detected by SNIPPY between the Illumina reads and its corresponding assembly were excluded. The mapped reads from the BAM were sorted so that read names appeared sequentially before extracting the reads using bedtools v. 2.29.2 bamtofastq functionality. If the extracted read coverage depth was <40X it was excluded from further analysis. Reads were then assembled in the same manner as the unfiltered reads and samples were excluded if their assembly metrics did not meet the criteria above. AMR genes were predicted from each assembly using the Comprehensive Antibiotic Resistance Database (CARD)’s Resistance Gene Identifier (RGI) software v.5.2.0 and CARD reference data v.3.1.4^24^.

To generate a simulated metagenomic benchmarking dataset, a reproducible nextflow^25^ simulation workflow was used. The generated gold-standard WGS assemblies were randomly amplified following a log-normal distribution (μ = 1 σ = 2) to represent observed metagenomic species distributions^26^. Additional CARD (v3.1.4) AMR reference genes were randomly inserted into the contigs to ensure representation of the full canonical CARD database in the metagenome. ART v2.5.8^27^ was then used to simulate 2.49 million 250 bp paired-end reads from these sequences using the Illumina MiSeqV3 error profile. Finally, using pysam (v0.16.0.1)^27,28^ and bedtools (v2.30.0)^23^ labels were generated for each read with the RGI (v5.2.0) annotated AMR gene from which that read was simulated.

**Fig. 2:**
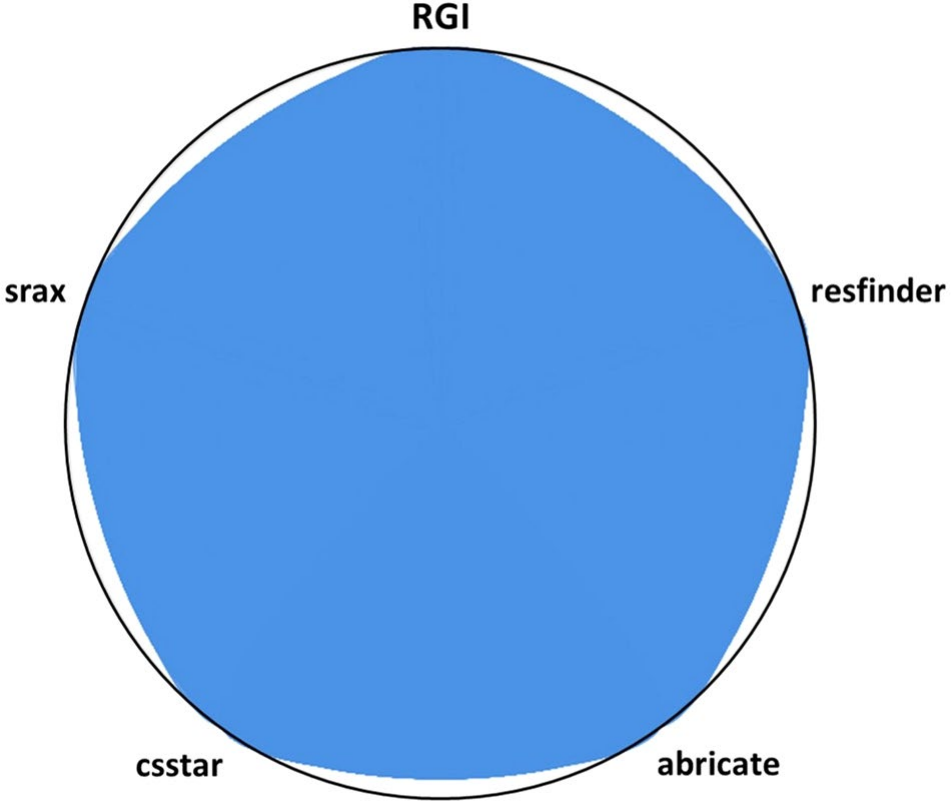
Radar plot showing 94 samples analyzed using hAMRonization workflow. There are 579 genes comparing presence or absence for all the 5 tools tested.

We selected RGI as it performs at par with other AMR tools evaluated using the hAMRonization workflow^29^. The hAMRonization workflow uses 12 different AMR tools to predict AMR genes in genomic data and produces a standard report to compare results across tools. Five of these 12 tools work with genomic reads, while the other 7 use assembled genomes. Analysis of 94 from 174 selected genomes was performed via the hAMRonization workflow using the 5 tools associated with assembled genome analysis. The RGI results produced were similar to the other 4 tools tested i.e., abricate, csstar, resfinder, and srax. The results are presented as a radar plot in Fig. 2 and available at Zenodo^30^.

## Data Records

The datasets are suitable for different AMR detection pipelines, as they provide assemblies using two different widely used assemblers in addition to mapped reads from the primary data used to generate the assembly for 174 bacterial genomes representing 22 distinct species (Table 1). To enable benchmarking of metagenomic AMR detection pipelines, these datasets also provide simulated metagenomic reads and a “perfect” metagenomic assembly derived from these 174 assemblies. Since it is possible for records to be updated in NCBI, we have included reads in the dataset to ensure that they can be consistently used. Due to the size of the data, we have split the dataset into assemblies, 6 batches of genomic reads, and a separate metagenomic dataset (including assemblies, reads, and label information).

**Table 1:** Taxonomic composition of the benchmarking dataset.

Organism	Sample Count
*Acinetobacter baumannii*	5
*Aeromonas veronii*	1
*Citrobacter freundii*	4
*Enterobacter asburiae*	2
*Enterobacter bugandensis*	1
*Enterobacter cancerogenus*	1
*Enterobacter cloacae*	3
*Enterobacter hormaechei*	10
*Enterobacter roggenkampii*	2
*Enterococcus faecium*	2
*Enterococcus* sp.	1
*Escherichia coli*	18
*Klebsiella aerogenes*	3
*Klebsiella oxytoca*	4
*Klebsiella pneumoniae*	56
*Kluyvera intermedia*	1
*Providencia stuartii*	1
*Pseudomonas aeruginosa*	6
*Salmonella enterica*	22
*Staphylococcus aureus*	30
*Staphylococcus lugdunensis*	1

The assemblies (which include closed, draft versions for raw and filtered datasets) are located at *Zenodo*^31^.

The mapped raw reads (BAM files) are located at *Zenodo*:

Mapped Read Sets – 1^32^

Mapped Read Sets – 2^33^

Mapped Read Sets – 3^34^

Mapped Read Sets – 4^35^

Mapped Read Sets – 5^36^

Mapped Read Sets – 6^37^

The simulated metagenomic data (reads, assemblies, labels, simulation configuration) are located at *Zenodo*^38^, with corresponding simulation workflow available at *Zenodo*^39^.

The corresponding metadata for all isolates can be found can be found at *Zenodo*^30^.

The Resistance Gene Identifier predictions can be found at Zenodo^30^. Note that each file name is the complete assemblies’ accession number.

## Technical Validation

The baseline data for the simulations were 100% completed genomes of ESKAPE pathogens, with accompanying FASTQ reads, all of which passed the National Center for Biotechnology Information curation process. The assembly and simulation software used to create benchmark metagenomic data sets have been previously validated in their own publications. As outlined in the Data Processing section, any assemblies or simulated reads not passing quality metrics were excluded.

## Usage Notes

Not used.

## Data Availability

Custom code (hAMRonization v1.0.3) was used to compare different AMR tools to predict AMR genes in genomic data and produce a standard report to compare results across tools (Fig. 2.).This code is available at Github^29^.
